# Formulation, evaluation and antimicrobial effect of herbal disinfectants

**DOI:** 10.6026/973206300210740

**Published:** 2025-04-30

**Authors:** Payala Vijayalakshmi, Sarada Vadlamani, Nitin Mohan

**Affiliations:** 1 Department of Microbiology, GITAM Institute of Medical Sciences and Research (Deemed to be University), Rushikonda, Visakhapatnam-530045, Andhra Pradesh, India; 2 Department of Community medicine, GITAM Institute of Medical Sciences and Research (Deemed to be University), Rushikonda, Visakhapatnam-530045, Andhra Pradesh, India; 3 Department of Microbiology, Central Laboratory, Institute of Medical Sciences and SUM Hospital -III, SOA (Deemed to be University), Sitalapalli, Narendrapur - 760007, Ganjam, Odisha, India

**Keywords:** Antimicrobial activity, fourier transform infrared spectroscopy (FTIR), herbal formulation, plants, scanning electron microscopy (SEM)

## Abstract

The cost-effective herbal disinfectant formulations from indigenously available plants with their potential antimicrobial activity against hospital-derived microbial flora are of interest. Hence, a total of six herbal disinfectant formulations were prepared with indigenously available plants and tested for the antimicrobial activity using a standard protocol. The results show that *Citrobacter koseri* isolated from the Ophthalmology ward showed the highest sensitivity to *Acacia nilotica* herbal disinfectant and the inhibition zone identified was 35mm higher than standard tested antibiotics. Chemical composition analysis of *Acacia nilotica*, *Moringa oleifera*, and *Senna alata* herbal formulations using qualitative and FTIR methods showed the presence of various bioactive compounds contributing to their antimicrobial activity. Thus, the promise for advancing infection control practices in addressing the challenges of antimicrobial resistance in healthcare settings is evident.

## Background:

Hospital floors are generally heavily contaminated at a mean bacterial count of 380 organisms/25cm^2^. Most bacteria on surfaces come from the skin flora of room occupants, with less than 1% being potential pathogens like *Staphylococcus aureus*. The bacterial count on surfaces stabilizes after a few hours, with cleaning by detergent reducing bacterial counts by 80% and disinfectants increasing that to over 95% [1]. However, recontamination occurs rapidly, often within 1-2 hours [2]. The transient reduction obtained does not justify the routine use of a disinfectant. Disinfection may still be required in areas of high risk or if the number of potential pathogens is thought to be high [3]. Routine disinfectant is necessary where there are high-risk areas or a large number of potential pathogens. Disinfectants like quaternary ammonium compounds and hypochlorite are common, but arguments against their overuse include rapid recolonization, environmental damage, occupational hazards by spills, development of resistance to chemical disinfectants, and cost [4-5]. Nowadays, herbal disinfectants are offered as an alternative and environment-friendly solution since many chemical disinfectants pose negative effects like skin irritation, disagreeable odor, and mainly in-house antimicrobial resistance towards many disinfectants exhibited by the hospital flora. The Spread of multidrug-resistant (MDR) microbial isolates in the hospital environment leads to strong resistance to chemical disinfectants that are routinely used for hospital mopping. Therefore, it is of interest to formulate safe and cost-effective herbal disinfectant formulations from indigenously available plants and to evaluate their potential antimicrobial activity against hospital-derived multidrug-resistant microbial pathogens causing HAIs.

## Methodology:

Study design: It is a hospital-based experimental study

Study place: The study was done in the Department of Microbiology and Central Research Laboratory, GITAM Institute of Medical Sciences and Research, Visakhapatnam.

Study period: The study was conducted for one year, from January 2023 to January 2024.

## Study procedure:

## Collection of plant material:

Six plants - *Ocimum sanctum*, *Azadirachta indica*, *Acacia nilotica*, Hibiscus rosa-sinensis, *Moringa oleifera* leaves, and *Senna alata* bark-were identified and confirmed by Andhra University's botanical department. The leaves were cleaned, sun-dried, powdered, and stored in a dry place. *Senna alata* bark powder was sourced from Visakhapatnam.

## Preparation of Herbal disinfectant formulations through solvent extraction method:

The plant powders were extracted using the soxhlet solvent extraction method with 250 ml of ethanol solvent. To prepare herbal formulations, 31ml of deionized water, 0.5ml of polysorbate, 0.7ml of triethanol amine, 2.3ml of glycerin,0.25g of isopropyl parabens (preservative),0.5% of perfume were added. The six prepared herbal formulations were stored in airtight high-density polyethylene (HDPE) containers.

## Surface sampling:

The swabs were collected from floors and walls of clinical departments, nonclinical departments, and OTs and ICUs in hospitals. Moistened swabs with direct plating are the most common method to sample the surfaces. Swabs were inoculated instantly into peptone water. Before the plating, each swab in the tube vortexed for 5-10 sec and then plated on appropriate media blood agar and MacConkey agar plates. Inoculated plates were incubated at 37°C for 24h, and the colonies were counted (CFU/cm^2^). Standard microbiological techniques like Gram staining and biochemical tests, including Indole, Citrate utilization, Catalase, Coagulase, Oxidase tests, Urease, and TSI, were used to identify microorganisms.

## Agar-well diffusion method:

Bacteria isolated in pure culture were tested for antimicrobial susceptibility using the agar-well diffusion method with six herbal formulations. A comparative study with standard antibiotics evaluated the antibacterial potency of commercial agents (5% alkyl dimethyl benzene ammonium, 2% silvicide) versus plant extracts, following CLSI guidelines with Mueller-Hinton agar plates and incubation at 37°C for 16-18 hours. Standard ATCC Microbial cultures were used as controls.

## Suspension test:

In the suspension test, 1 ml of test inoculum is added to 10 ml of herbal disinfectant, with controls using ethanol or water. Contact times of 10, 20 and 30 minutes are tested, followed by dilution and incubation on blood agar at 37°C for 24 hours.

## Phytochemical screening:

Phytochemical testing was conducted on six plant samples to identify secondary metabolites such as alkaloids, flavonoids, polyphenols, terpenoids, saponins, tannins, glycosides, quinones, coumarins, and phenolic compounds. Qualitative tests, including Mayer's, Wiefferering, frothing, Ferric chloride, Salkowski, Pew's and 3, 5-dinitro benzoic acid tests, were performed. To confirm the presence of these metabolites, Fourier Transform Infrared Spectroscopy (FTIR) was used to analyze the functional groups in the plant extracts, identifying peaks associated with antimicrobial activity. The leaf morphology was studied using Field Emission Scanning Electron Microscopy (FE-SEM) with Energy Dispersive Spectroscopy (EDS) to examine spores' structure. FE-SEM provides detailed microstructure images, while EDS identifies the chemical elements present. This combination of methods aids in identifying antimicrobial phytochemicals and their potential mechanisms against microbes.

## Results:

## Preparation of herbal disinfectants:

Figure 1 (see PDF) represents the six herbal preparations prepared as mentioned in the methodology section. Microorganisms including *Citrobacter koseri*, *Staphylococcus aureus*, *Pseudomonas aeruginosa*, Coagulase-negative Staphylococci (CONS), and *Klebsiella pneumoniae* were isolated from various hospital areas. *C. koseri* from the Ophthalmology Ward showed sensitivity to meropenem and ciprofloxacin but resistance to cefepime and ceftazidime/Clavulanic acid (CAC). *S. aureus* and *K. pneumoniae* from the General Surgery and Dermatology wards showed sensitivity to cefepime and ciprofloxacin, with resistance to CAC. *P. aeruginosa* from the Ophthalmology OT and other locations displayed sensitivity to PIP/TAZ and ciprofloxacin but resistance to CAC and meropenem (MRP). Detailed susceptibility patterns are presented in Table 1 and Table 2 and Figure 2a and Figure 2b (see PDF). *Citrobacter koseri* from the Ophthalmology ward showed the highest sensitivity to *Acacia nilotica* herbal disinfectant, with a 35mm inhibition zone, surpassing ciprofloxacin (25mm) and meropenem (23mm). *Klebsiella pneumoniae* from the Gynaecology ward had a 33mm inhibition zone to *Acacia nilotica*, outperforming cefepime (27mm). *Senna alata* disinfectant showed the best antimicrobial activity against Coagulase negative Staphylococci, with a 20mm inhibition zone, matching gentamicin and amikacin as shown in Table 3. Although the other four herbal disinfectants prepared with moringa leaves, Hibiscus, Neem, and Ocimum showed good antimicrobial activity, they were less effective than tested antibiotics (Figure 3 - see PDF).

## Results of Suspension test:

After 10-30 minutes of exposure to six disinfectant solutions, no bacterial growth was observed on nutrient agar for *Acacia nilotica*, *Moringa oleifera*, and *Senna alata* at 20 minutes, indicating good antimicrobial activity (Figure 4 - see PDF).

## Phytochemical screening:

All six herbal formulations tested positive for alkaloids, flavonoids, terpenoids, saponin, glycosides, quinines, and phenolic compounds, except for tannins in Moringa. Fourier-Transform Infrared Spectroscopy confirmed the presence of functional groups in secondary metabolites, identifying compounds like alcoholic, alkane/alkene, and polymeric compounds based on the graph peaks. Figure 5 (see PDF) shows the absorption spectrum of *Acacia nilotica*. Key peaks include 3290 cm-1 for hydroxy compounds (O-H stretch), 2918 cm-1 for aliphatic compounds (C-H stretch), 2850 cm-1 for proteins/lipids (C-H stretch), 1732 cm-1 for ketones (C=O stretch), and 1317 cm-1 for aromatic primary amines (CN stretch), among others.

Figure 6 (see PDF) shows *Senna alata*'s absorption spectrum. Key peaks include 3271 cm-1 (hydroxy), 2929 cm-1 (aliphatic), 1605 cm-1 (ketones), 1314 cm-1 (aromatic primary amines), and 1031 cm-1 (aliphatic fluoro compound). Figure 7 (see PDF) displays *Moringa oleifera*'s absorption spectrum with key peaks: 3287 cm-1 (hydroxy), 2918 cm-1 (aliphatic), 2849 cm-1 (proteins/lipids), 1594 cm-1 (ketones), 1395 cm-1 (phenols/alcohols), and 1016 cm-1 (aliphatic fluoro compound)

## Scanning electron microscopy:

SEM analysis of *Acacia nilotica* shows an abundance of spores, enhancing nutrient absorption and preventing microbial entry. If microbes enter, the spores can break their chain, making them susceptible (Figure 8 - see PDF).

## Discussion:

Infection prevention is crucial in healthcare, and effective disinfection is essential for maintaining cleanliness. While traditional chemical disinfectants are commonly used, concerns about their adverse effects and the rise of drug-resistant microbes have led to interest in herbal alternatives. The findings of this research are novel, as no previous studies have addressed the preparation of herbal formulations of six plant extracts as disinfectants to inhibit the hospital microbial flora. The research outlines the collection, extraction, and formulation of plant materials into herbal disinfectants. These formulations underwent rigorous evaluation, including surface sampling, antimicrobial susceptibility testing, phytochemical screening, and chemical composition analysis. Established techniques like the agar-well diffusion method and Fourier transform infrared spectroscopy (FTIR) were employed to gain insights into the antimicrobial properties and chemical composition of the herbal disinfectants. The findings indicate significant antimicrobial activity in certain herbal formulations, surpassing the effectiveness of standard antibiotics against specific microbial strains. Among the six herbal formulations tested, two prepared with *Acacia nilotica* and *Senna alata* showed good antimicrobial activity against the hospital flora. Chemical composition analysis by qualitative and FTIR methods of *Acacia nilotica*, *Moringa oleifera*, and *Senna alata* herbal formulations identified bioactive compounds such as tannins, terpenoids, anthraquinones, saponins, flavonoids, alkaloids, glycosides in the herbal formulations, likely contributing to their antimicrobial activity. This aligns with prior research highlighting the antimicrobial potential of plant-derived compounds. Gurjinder *et al.* [6] reported the antimicrobial activity of crude extract of *Acacia nilotica* towards *B. subtilis* and the zone of inhibition was 22mm. However, the present research yields better results on the antimicrobial activity of *A. nilotica*. The *Citrobacter koseri* isolated from the Ophthalmology ward showed the highest sensitivity to *Acacia nilotica* herbal disinfectant with zone of inhibition of 35mm. This zone size was comparatively more significant than the one obtained with antibiotic ciprofloxacin and meropenem (25mm). *Klebsiella pneumoniae* species isolated from the Gynaecology ward showed the highest sensitivity to herbal disinfectant prepared with *Acacia nilotica* leaves. The zone of inhibition is 33mm, which is comparatively more significant than the zone of inhibition obtained with antibiotic cefepime, which is 27mm. Chandrasekhar *et al.* [7] showed the maximum inhibitory zone size of 22mm of *A. nilotica* toward *Streptococcus mutans*. Deshpande and Kadam *et al.* [8] found that ethanolic extracts of *A.nilotica* were effective in inhibiting the growth of Streptococcus mutans, and the zone size was measured as 31mm. Elamary *et al.* [9] studied the efficacy of *A. nilotica*aqueous extract on survival and biofilm-producing MDR pathogens - *E.coli*, *K. pneumoniae*, Proteus mirabilis, *Pseudomonas aeruginosa* and *Acinetobacter baumanii* isolated from patients suffering with UTI syndrome. Revathi *et al.* [10] and Abeer *et al.* [11] showed the opulent antimicrobial activity of ethanolic extract of *A. nilotica* against *Staphylococcus aureus*. Herbal disinfectant formulation of *Senna alata* showed the best antimicrobial susceptibility towards Coagulase negative Staphylococci isolated from gynecology OT, and the zone of inhibition measured was 20mm. The zone size equals the standard antibiotics zone sizes of gentamicin and amikacin. Karthika *et al.* [12] evaluated the phytochemical analysis of *Senna alata* leaves and found that aqueous leaf extracts of *Senna alata* showed good antimicrobial activity against *S. typhi* and *B. subtilis*. The zone sizes measured were 28mm for both cultures. Similarly, Doughari *et al.* [13] reported that the antimicrobial activity of *Senna alata* had influenced the growth of *S. aureus* and *S. pyogenes*. Similar to the findings of the current study, Chowdary *et al.* showed good antimicrobial activity of Costus igneus against hospital derived *Pseudomonas aeruginosa* [14] and Pandya *et al.* showed *Terminalia chebula* showed good antimicrobial activity against Escherichia coli and *Staphylococcus aureus* [15]. It was concluded that the above research provides a scientific basis for *A. nilotica* and *S. alata* herbal formulations to be used as local herbal disinfectants.

## Conclusion:

The potential of herbal disinfectants as sustainable, cost-effective alternatives to chemicals in hospitals settings is highlighted. Further studies on antimicrobial properties, formulation standardization and practical feasibility, addressing challenges like geographic variations, scalability, and regulatory acceptance to enhance infection control and combat antimicrobial resistance is needed.

## Declaration of Helsinki:

The author declared that the study was not involved with human subjects or animals subjects.

## Figures and Tables

**Table 1:** Isolated microorganisms by surface sampling technique

**Study area**	**Microorganisms isolated**
Ophthalmology ward	*Citrobacter koseri*
General surgery ward	*Staphylococcus aureus*
Psychiatric ward, ENT ward, ENT OT, Library, Skill lab, Biochemistry, Physiology, RTPCR, Central, Research lab, Community medicine, Maternity, TBCD	No growth
Dermatology ward	*Klebsiella pneumoniae*
Gynaecology ward	*Klebsiella pneumoniae*
Ophthalmology OT	*Pseudomonas aeruginosa*
**Gynaecology OT**	**CONS**
Anatomy	*Pseudomonas aeruginosa*
Causality	*Pseudomonas aeruginosa*

**Table 2:** Antimicrobial susceptibility of isolated microorganisms by surface sampling

**Ward/OT/ Department (Floors)**	**Possible Isolate**	**Antibiotics (Ressitant/Intermediate /Sensitive) Zone of inhibition in mm**
Ophthalmology ward	*Citrobacter koseri*	Meropenem: 23mm (S) Ciprofloxacin: 25mm (S) Gentamicin:18mm (S) PIP/TAZ: 19mm (IS) Cefepime: no zone (R) Ceftazidime/CA: no zone (R) Amikacin: 18mm (S)
General surgery (Female ward)	*Staphylococcus aureus*	Meropenem: 22mm (S) Ciprofloxacin: 23mm (S) Gentamicin:17mm (S) PIP/TAZ: 15mm (IS) Cefepime: 25mm (S) Ceftazidime/CA: no zone (R) Amikacin: 17mm (S)
Dermatology ward	*Klebsiella pneumoniae*	Meropenem: 23mm (S) Ciprofloxacin: 25mm (S) Gentamicin:18mm (S) PIP/TAZ: 20mm (IS) Cefepime: 28mm (S) Ceftazidime/CA: no zone (R) Amikacin: 18mm (S)
Gynaecology ward	*Klebsiella pneumoniae*	Meropenem: 23mm (S) Ciprofloxacin: 23mm (S) Gentamicin:17mm (S) PIP/TAZ: 19mm (IS) Cefepime: 27mm (S) Ceftazidime/CA: no zone (R) Amikacin: 18mm (S)
Ophthalmology OT	*Pseudomonas aeruginosa*	Meropenem: 17mm (R) Ciprofloxacin: 23mm (S) Gentamicin:18mm (S) PIP/TAZ: 22mm (S) Cefepime: no zone (R) Ceftazidime/CA: no zone (R) Amikacin: 19mm (S)
Gynaecology OT	Coagulase-negative staphylococci	Meropenem: 18mm (R) Ciprofloxacin: 24mm (S) Gentamicin:20mm (S) PIP/TAZ: 21mm (S) Cefepime: 29mm (S) Ceftazidime/CA: 9mm (R) Amikacin: 20mm (S)
Anatomy	*Pseudomonas aeruginosa*	Meropenem: 18mm (R) Ciprofloxacin: 25mm (S) Gentamicin:21mm (S) PIP/TAZ: 23mm (S) Cefepime: 28mm (S) Ceftazidime/CA: no zone (R) Amikacin: 18mm (S)
Causality	*Pseudomonas aeruginosa*	Meropenem: 10mm (IS) Ciprofloxacin: 26mm (S) Gentamicin:20mm (S) PIP/TAZ: 22mm (S) Cefepime: 32mm (S) Ceftazidime/CA: 8mm (R) Amikacin: 20mm (S)

**Table 3:** Antimicrobial susceptibility with plant extracts

**Study site**	**Microorganism isolated**	**Zone of inhibition in mm *Moringa oleifera***	**Zone of inhibition in mm *Hibiscus rosasinensis***	**Zone of inhibition in mm *Azadirachta indica***	**Zone of inhibition in mm *Senna alata***	**Zone of inhibition in mm *Ocimum sanctum***	**Zone of inhibition in mm *Acacia nilotica***
Ophthalmology ward	*Citrobacter koseri*	8mm	12mm	9mm	14mm	11mm	35mm
General surgery (Female ward)	*Staphylococcus aureus*	9mm	14mm	10mm	10mm	16mm	13mm
Dermatology ward	Klebsiella pneumonia	7mm	13mm	11mm	15mm	10mm	11mm
Gynaeic ward	Klebsiella pneumonia	7mm	13mm	8mm	14mm	9mm	33mm
Opthal OT	*Pseudomonas aeruginosa*	No zone	11mm	12mm	14mm	10mm	19mm
Gyn OT	Coagulase-negative staphylococci	No zone	12mm	7mm	20mm	9mm	17mm
Anatomy	*Pseudomonas aeruginosa*	10mm	14mm	11mm	9mm	16mm	14mm
Causality	*Pseudomonas aeruginosa*	18mm	15mm	10mm	11mm	15mm	16mm
